# Screening for hypertrophic cardiomyopathy: a cost analysis of echocardiography, cardiac magnetic resonance and genetic testing

**DOI:** 10.1186/1532-429X-11-S1-O70

**Published:** 2009-01-28

**Authors:** Thomas H Hauser, Martin S Maron, Warren J Manning

**Affiliations:** Beth Israel Deaconess Medical Center, Boston, MA USA

**Keywords:** Diagnostic Accuracy, Genetic Testing, Cardiovascular Magnetic Resonance, Cardiac Magnetic Resonance, Sudden Cardiac Death

## Introduction

Hypertrophic cardiomyopathy (HCM) is the most common heritable cardiomyopathy and is the leading cause of sudden cardiac death early in life. Screening of first degree relatives for HCM phenotype is recommended, typically with serial echocardiography (Echo). Recently, cardiovascular magnetic resonance (CMR) and genetic testing (GT) have emerged as additional screening strategies. Up to 5% of patients with HCM on CMR have normal Echo findings. GT has been proposed as a method for eliminating unnecessary diagnostic imaging tests in genotype negative family members in families identified to have a disease causing mutation, present approximately 50% of the time. Because large, long-term trials to compare these methods are not feasible, modeling of costs and outcomes can provide insight into the potential costs and benefits of various screening strategies.

## Purpose

The objective of this study is to evaluate the costs and benefits of screening strategies of HCM using Echo, CMR and GT.

## Methods

We developed a Markov model to evaluate 5 screening strategies: Echo, CMR, Echo and GT, CMR and GT, and Echo, CMR and GT. All screening strategies were designed based on standard guidelines with first testing at age 13, with imaging with Echo and/or CMR every 2 years until age 25 and every 7 years until age 50. GT was performed once only at the start of screening. The model was tested using a theoretical cohort of 1000 probands with an average of 4 first degree relatives that required testing. The base case assumed an Echo cost of $650, CMR cost of $800, and GT cost of $3000. The penetrance of HCM was modeled such that, of those destined to have HCM, 90% of screened first degree relatives would have phenotypic HCM by age 25 with the remaining 10% evident by age 50. Echo was assumed to have a 5% error rate compared to CMR. The analysis was performed from the societal perspective using constant 2008 dollars. A 3% discount rate was used.

## Results

**Figure 1:**
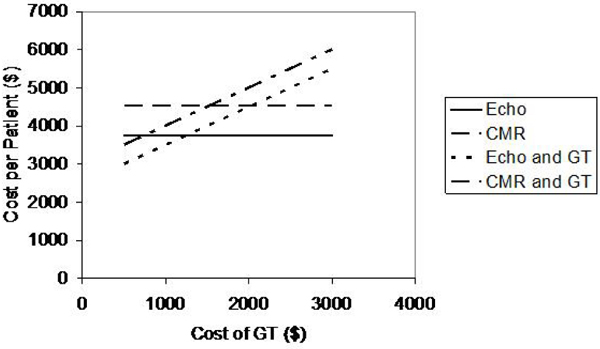
**Cost per patient for HCM screening**.

GT resulted in increased costs with no improvement in diagnostic accuracy when paired with either Echo or CMR. Echo was the least expensive strategy with a cost per patient screened of $3754, but did not identify phenotypic HCM in 6%. CMR cost $4546 per patient screened and did not identify 1% of patients with phenotypic HCM. The incremental cost for each additional diagnosis of HCM with CMR was $15,840. The addition of Echo to CMR increases costs without any increase in diagnostic accuracy. Sensitivity analysis showed that the cost of GT had a large influence on the analysis. The break even cost for GT was $1243 when used with Echo and $1530 when used with CMR (figure 1).

## Conclusion

In this model of screening for HCM, GT increased costs without improving outcome. Screening with CMR identified a larger proportion of patients with HCM with an associated increase in cost.

